# Effects of Melatonin Supplementation on Hormonal, Inflammatory, Genetic, and Oxidative Stress Parameters in Women With Polycystic Ovary Syndrome

**DOI:** 10.3389/fendo.2019.00273

**Published:** 2019-05-14

**Authors:** Mehri Jamilian, Fatemeh Foroozanfard, Naghmeh Mirhosseini, Elham Kavossian, Esmat Aghadavod, Fereshteh Bahmani, Vahidreza Ostadmohammadi, Mersedeh Kia, Tahereh Eftekhar, Elnaz Ayati, Mostafa Mahdavinia, Zatollah Asemi

**Affiliations:** ^1^Traditional and Complementary Medicine Research Center, Arak University of Medical Sciences, Arak, Iran; ^2^Gametogenesis Research Center, Kashan University of Medical Sciences, Kashan, Iran; ^3^School of Public Health, University of Saskatchewan, Saskatoon, SK, Canada; ^4^Research Center for Biochemistry and Nutrition in Metabolic Diseases, Kashan University of Medical Sciences, Kashan, Iran; ^5^Department of Midwifery, Gorgan Branch, Islamic Azad University, Gorgan, Iran; ^6^Reproductive Health Research Center, Tehran University of Medical Science, Tehran, Iran; ^7^Department of Gynecology and Obstetrics, School of Medicine, Tehran University of Medical Sciences, Tehran, Iran; ^8^Department of Dermatology, Razi Hospital, Tehran University of Medical Sciences, Tehran, Iran

**Keywords:** melatonin, hormonal profiles, inflammatory markers, polycystic ovary syndrome, oxidative stress

## Abstract

**Purpose:** The aim of the current study was to evaluate the effect of melatonin administration on clinical, hormonal, inflammatory, and genetic parameters in women with polycystic ovarian syndrome (PCOS).

**Methods:** The present randomized, double-blinded, placebo-controlled clinical trial was conducted among 56 patients with PCOS, aged 18–40 years old. Subjects were randomly allocated to take either 5 mg melatonin supplements (*n* = 28) or placebo (*n* = 28) twice a day for 12 weeks.

**Results:** Melatonin administration significantly reduced hirsutism (β −0.47; 95% CI, −0.86, −0.09; *P* = 0.01), serum total testosterone (β −0.11 ng/mL; 95% CI, −0.21, −0.02; *P* = 0.01), high-sensitivity C-reactive protein (hs-CRP) (β −0.61 mg/L; 95% CI, −0.95, −0.26; *P* = 0.001), and plasma malondialdehyde (MDA) levels (β −0.25 μmol/L; 95% CI, −0.38, −0.11; *P* < 0.001), and significantly increased plasma total antioxidant capacity (TAC) levels (β 106.07 mmol/L; 95% CI, 62.87, 149.28; *P* < 0.001) and total glutathione (GSH) (β 81.05 μmol/L; 95% CI, 36.08, 126.03; *P* = 0.001) compared with the placebo. Moreover, melatonin supplementation downregulated gene expression of interleukin-1 (IL-1) (*P* = 0.03) and tumor necrosis factor alpha (TNF-α) (*P* = 0.01) compared with the placebo.

**Conclusions:** Overall, melatonin administration for 12 weeks to women with PCOS significantly reduced hirsutism, total testosterone, hs-CRP, and MDA, while increasing TAC and GSH levels. In addition, melatonin administration reduced gene expression of IL-1 and TNF-α.

**Clinical Trial Registration:** www.irct.ir, identifier IRCT2017082733941N9, Available online at: https://www.irct.ir/trial/26051

## Introduction

Polycystic ovarian syndrome (PCOS) is a highly prevalent endocrinopathy affecting up to 10% of premenopausal women and essentially characterized by hyperandrogenism, reproductive abnormalities, and ovulatory dysfunction (1). Although the exact pathophysiology of PCOS is not yet fully recognized, evidence suggests that hyperandrogenism plays a central role in this syndrome (2, 3). Hyperandrogenism might occur due to the inflammatory response of the abnormal ovarian theca cells to free oxygen radicals (4). Furthermore, prior studies have indicated that increased oxidative stress and elevated inflammatory markers such as C-reactive protein (CRP) in women with PCOS are correlated with obesity, insulin resistance, and an increased risk of cardiovascular disease (CVD) (5, 6). The majority of women with PCOS suffer from hirsutism, acne, oligomenorrhea, and androgenic alopecia, which impair the quality of life of these women (7).

Melatonin (N-acetyl-5-methoxytryptamine) is secreted by the pineal gland (8). The circadian pattern of its secretion is regulated by the biological clock within the hypothalamic suprachiasmatic nucleus (9). Some of the major functions of rhythmic secretion of melatonin in blood include regulation of sleep, modulation of circadian rhythms (10), and involvement in the body's immune response (11). Melatonin is also a powerful endogenous antioxidant (12), and its supplementation has radical-scavenging and anti-inflammatory actions (13, 14). Women with PCOS are often sub-fertile secondary to ovulatory dysfunction, impaired quality of oocyte, and low endometrial receptivity (15). *In vitro* fertilization (IVF) is recommended as an assisted reproduction technology for infertile patients with PCOS when patients do not respond to ovulation induction agents (16). Current evidence suggests the involvement of melatonin in ovarian physiology including ovulation, follicular development, luteal function, and oocyte maturation. Furthermore, melatonin deficiency seems to be involved in the pathophysiology of PCOS (17). It has been shown that melatonin is an efficient predictor of oocyte quality and IVF outcomes: indeed, its high concentration correlates positively with a satisfactory quality of oocytes (18). On the other hand, intra-follicular melatonin level is significantly reduced in patients with PCOS (18). In addition, Jain et al. (19) demonstrated that nocturnal melatonin levels in women with PCOS are higher than in control subjects. Elevated nighttime melatonin levels in PCOS have been explained by a feedback mechanism due to its decreased concentration in the ovarian follicles (20). So, women with PCOS who have high oxidative stress are synthesizing more melatonin, probably in an effort to eliminate extra free radicals (21). High melatonin level in the ovarian follicle fluid is pivotal for ovulation, follicular growth, and quality of oocyte, whereas reduced melatonin level in follicular fluid may be responsible for decreased oocyte quality and anovulation in these women (22). In a recent study, melatonin and myo-inositol co-supplementation significantly improved embryo and oocyte quality in women with PCOS who were candidates for IVF, when compared with myo-inositol alone (23). In addition, treatment with melatonin and myo-inositol significantly improved ovarian stimulation protocols and pregnancy outcomes in women who failed to conceive in previous IVF cycles because of poor quality of the oocyte (24).

There is promising evidence showing the importance of melatonin supplementation in patients with PCOS who are candidates for IVF. However, whether melatonin has direct benefits on hirsutism, hormonal profiles, and biomarkers of inflammation/oxidative stress in these patients has not been assessed yet. The present study was, therefore, carried out to evaluate the effects of melatonin administration on clinical status, hormonal profiles, oxidative stress biomarkers, inflammatory factors, and gene expression related to inflammation in women diagnosed with PCOS who were candidates for IVF.

## Materials and Methods

### Trial Design and Participants' Characteristics

This randomized, double-blinded, placebo-controlled trial was registered in the Iranian website for registration of clinical trials (http://www.irct.ir: IRCT2017082733941N9) and followed the Declaration of Helsinki and Good Clinical Practice guidelines. This trial was conducted among 56 women aged 18–40 years old diagnosed with PCOS, based on the Rotterdam criteria (25), who had been referred to the Kosar Clinic in Arak, Iran, between August and December 2017. The study protocol was approved by the ethics committee of Arak University of Medical Sciences (AUMS). Written informed consent was obtained from all participants prior to the intervention. Exclusion criteria were as follows: pregnancy, breastfeeding, sleeping disorders, adrenal hyperplasia, androgen-secreting tumors, hyperprolactinemia, thyroid dysfunction, and/or diabetes at enrollment.

### Supplementation

Initially, patients were randomly divided into two groups to take either melatonin capsules (2 × 5 mg/day) (*n* = 28) or placebo (*n* = 28) for 12 weeks. Melatonin and the placebo were manufactured by Zahravi Pharmaceutical Company (Tabriz, Iran) and Barij Essence Pharmaceutical Company (Kashan, Iran), respectively. The placebo capsules were matched in color, shape, size, packaging, smell, and taste with the melatonin capsules. Due to a lack of evidence regarding the appropriate dosage of melatonin for patients with PCOS, we used the following dose of melatonin based on a previous published study in diabetic patients with coronary heart disease (CHD) (26). All individuals were taking metformin tablets at an initial dose of 500 mg, which was increased in a stepwise manner during the first 3 weeks to a total of 1,500 mg/day (27). In addition, all individuals were taking 10 mg/day medroxyprogesterone tablet from the 15th until the 25th day of the menstrual cycle. Throughout the study, the consumption of melatonin supplements and placebos was assessed by asking subjects to return the medication containers as well as sending a brief daily cell phone reminder to take the supplements. Patients were instructed to have their regular physical activity and not take any extra nutritional supplements during the 12-week trial. All patients completed a 3-day food record (a weekend and 2 weekdays) at the baseline of the study, week 3, week 6, week 9, and the end of the intervention. Daily macro- and micronutrient intakes were calculated by analyzing 3-day food records, using nutritionist IV software (First Databank, San Bruno, CA).

### Assessment of Outcomes

Malondialdehyde (MDA) change was considered as the primary outcome. Serum total testosterone, sex hormone–binding globulin (SHBG), high-sensitivity C-reactive protein (hs-CRP), plasma nitric oxide (NO), total antioxidant capacity (TAC), total glutathione (GSH), hirsutism, and gene expression related to inflammation were recognized as the secondary outcomes.

### Clinical Measures

Hirsutism was assessed using a modified Ferriman–Gallwey (mFG) scoring system as 9 body areas, including the upper lip, chin, chest, upper abdomen, lower abdomen, thighs, back, arm, and buttocks, were investigated for hair, from 0 (no hair) to 4 (frankly virile) (28, 29).

### Biochemical Assessment

Fasting blood samples (15 ml) were collected at baseline and the end of the intervention at the Arak reference laboratory. Blood samples were immediately centrifuged (Hettich D-78532, Tuttlingen, Germany) at 3,500 rpm for 10 min to separate serum. Then, the samples were stored at −80°C at the AUMS reference laboratory until further analysis. Whole blood samples were used to check gene expression; serum samples to evaluate total testosterone, SHG, and hs-CRP; and plasma samples to measure NO, TAC, GSH, and MDA. Serum total testosterone and SHBG were measured using commercial validated kits (DiaMetra, Milano, Italy) with inter- and intra-assay coefficient variances (CVs) below 6.5%. Serum hs-CRP concentrations were quantified using a commercial ELISA kit (LDN, Nordhorn, Germany) with inter- and intra-assay CVs below 7%. The plasma NO was measured using the Griess method (30), TAC concentrations using the Benzie and Strain method (31), GSH using the Beutler method (32), and MDA concentrations of thiobarbituric acid–reactive substances using a spectrophotometric test with CVs below 5%. Measurements of hormonal profiles and biomarkers of inflammation and oxidative stress were performed in a blinded fashion, in duplicate, in pairs (pre/post-intervention) at the same time, in the same analytical run, and in random order to reduce systematic error and inter-assay variability.

### Isolation of Lymphocyte, RNA Extraction, and cDNA Synthesis

Whole blood samples were collected in anticoagulant EDTA tubes. Lymphocytes were isolated using 50% Percoll solution (Sigma-Aldrich, Dorset, UK) gradient by centrifugation for 20 min at 3,000 rpm at 4°C (33). Total RNA was extracted using the acid guanidinium–phenol–chloroform procedure and the application of RNX™-plus reagent (Cinnacolon, Tehran, Iran) following manufacturer's instructions. RNAs were treated with DNase I (Fermentas, Lithuania) for the elimination of any genomic DNA contamination. Concentration, integration, and purity of RNA samples were determined by spectrometry and gel electrophoresis. Three micrograms of total RNA was used for cDNA synthesis with random hexamer and oligo(dT)18 primers through RevertAid™ Reverse Transcriptase (Fermantase, Canada) in a total of 20-μl reaction mixture (33).

### Real-Time PCR Analysis

Appropriate primers were designed for interleukin-1 (IL-1), IL-8, tumor necrosis factor-α (TNF-α), transforming growth factor beta (TGF-β), and vascular endothelial growth factor (VEGF), and glyceraldehyde-3 phosphate dehydrogenase as the internal control (housekeeping gene; Table 1). Quantitative real-time PCR was performed using LightCycler® 96 sequence detection systems (Roche Diagnostics, Rotkreuz, Switzerland) with the application of 4 μl of 5 × EVA GREEN I master mix (Salise Biodyne, Japan), 10 ng cDNA, and 200 nM each of forward and reverse primers in a final volume of 20 μl. The PCR was performed through the following instruction: an initial denaturation at 95°C for 10 min, followed by 40 cycles of denaturation at 95°C for 10 s, annealing at 54–62.1°C for 15 s, and extension at 72°C for 30 s. The specificity of PCR products was evaluated by 1.5% agarose gel electrophoresis and melting curve analysis. All experiments were performed at least in triplicate.

**Table 1:** Specific primers used for real-time quantitative PCR.

**Gene**	**Primer**	**Product size (bp)**	**Annealing temperature (C)**
GAPDH	F: AAGCTCATTTCCTGGTATGACAACG	126	61.3
	R: TCTTCCTCTTGTGCTCTTGCTGG		
IL-1	F: GCTTCTCTCTGGTCCTTGG	174	56
	R: AGGGCAGGGTAGAGAAGAG		
IL-8	F: GCAGAGGGTTGTGGAGAAGT	150	56
	R: ACCCTACAACAGACCCACAC		
TNF-α	F: GTCAACCTCCTCTCTGCCAT	188	52
	R: CCAAAGTAGACCTGCCCAGA		
TGF-β	F: TTGAGACTTTTCCGTTGCCG	227	56
	R: CGAGGTCTGGGGAAAAGTCT		
VEGF	F: CTTCTGAGTTGCCCAGGAGA	216	54
	R: CTCACACACACACAACCAGG		

*GAPDH, glyceraldehyde-3-phosphate dehydrogenase; IL-1, interleukin-1; IL-8, interleukin-8; TNF-α, tumor necrosis factor alpha; TGF-β, transforming growth factor beta; VEGF, vascular endothelial growth factor*.

### Sample Size

To determine sample size, we used a randomized clinical trial (RCT) sample-size formula where type I (α) and type II errors (β) were 0.05 and 0.20 (power = 80%). Based on a previous RCT (26), we used a standard deviation of 0.30 μmol/L and a difference in mean (*d*) of 0.24 μmol/L, considering MDA as the key variable. The calculation indicated that 25 participants were required in each group. Assuming a dropout of five patients per group, the final sample size was determined to be 30 participants per group.

### Randomization

Study participants were randomized using computer-generated random numbers. Randomization and allocation were concealed from both researchers and patients until the completion of final analyses. The randomized allocation sequence, enrollment of participants, and allocation to interventions were conducted by a trained staff at the clinic. Another person, who was not involved in the trial and not aware of random sequences, assigned the subjects to the numbered bottles of capsules.

### Statistical Analyses

The Kolmogorov–Smirnov test was done to determine the normality of data. Differences in dietary intakes and gene expression were detected using independent-sample *t*-tests between treatment groups. Multiple linear regression models were used to assess the treatment effects on study outcomes, after adjusting for confounding parameters including age and BMI. The significance of the treatment effects was presented as the mean differences with 95% confidence interval. *P*-values < 0.05 were considered statistically significant. All statistical analyses were done using the Statistical Package for Social Science version 18 (SPSS Inc., Chicago, Illinois, USA).

## Results

**Figure 1:**
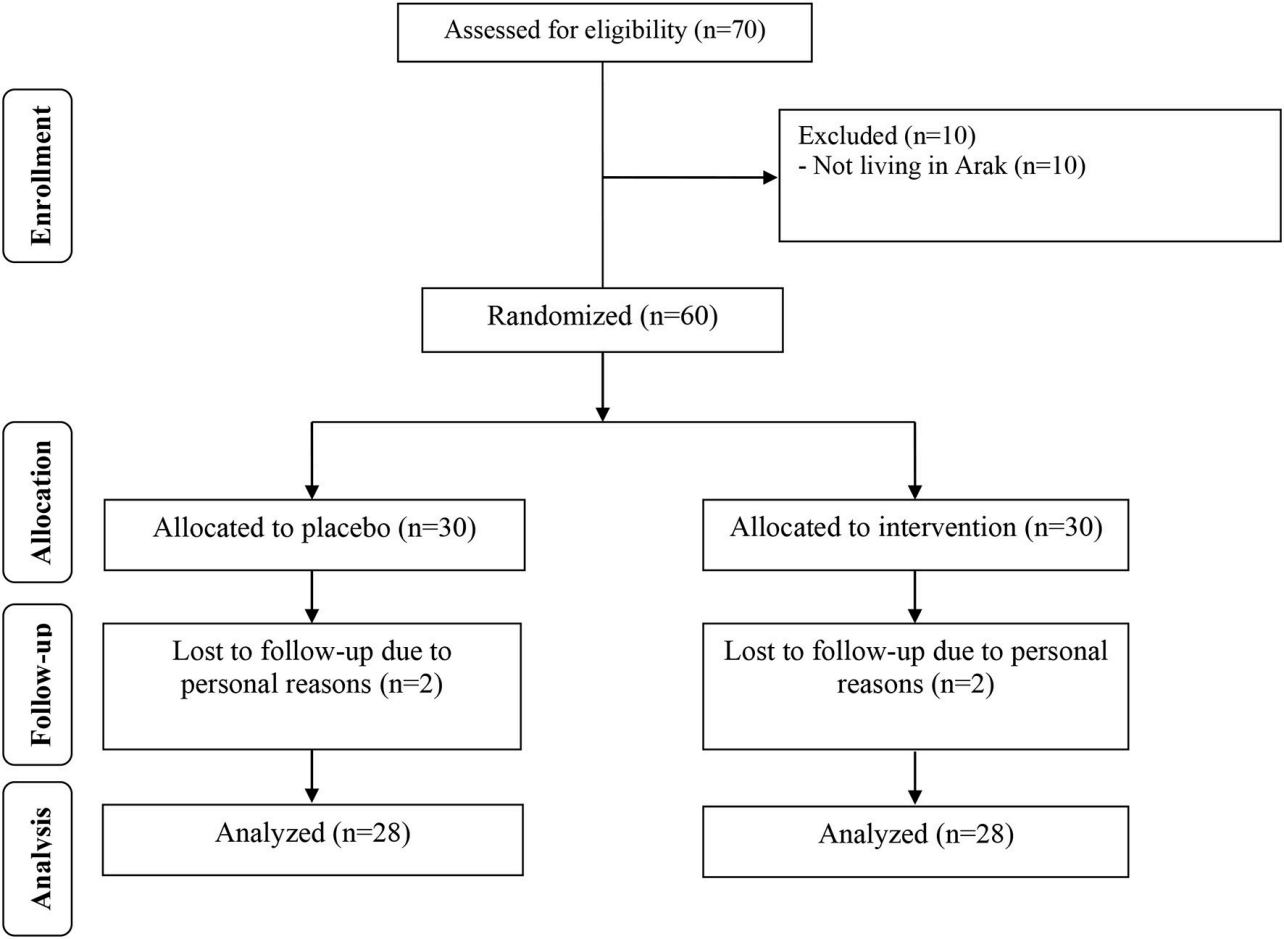
Summary of patient flow diagram.

During the study period, four participants dropped out of the study due to personal reasons (two participants in each group; Figure 1). Fifty-six participants [placebo (*n* = 28) and melatonin (*n* = 28)] completed the trial.

Anthropometric measures were not statistically different between intervention groups (Table 2).

**Table 2:** General characteristics of study participants^a^.

	**Placebo group (*n* = 28)**	**Melatonin group (*n* = 28)**	***P*^**b**^**
Age (y)	28.3 ± 2.3	28.7 ± 2.1	0.76
Height (cm)	160.0 ± 2.9	161.0 ± 2.5	0.30
Weight at study baseline (kg)	74.8 ± 8.9	75.1 ± 11.0	0.90
Weight at end of trial (kg)	74.5 ± 8.9	74.7 ± 10.9	0.92
Weight change (kg)	−0.3 ± 0.9	−0.4 ± 1.1	0.79
BMI at study baseline (kg/m^2^)	29.2 ± 3.5	29.1 ± 4.6	0.92
BMI at end of trial (kg/m^2^)	29.1 ± 3.4	29.0 ± 4.6	0.91
BMI change (kg/m^2^)	−0.1 ± 0.3	−0.1 ± 0.4	0.84

a *Data are means ± SDs*.

b *Obtained from independent t-test*.

There was no statistically significant difference in dietary macro- and micro-nutrient intakes between the melatonin and placebo groups (Table 3).

**Table 3:** Mean dietary intakes of study participants at baseline, week 3, week 6, and week 9, and the end of the intervention.

	**Placebo group (*n* = 28)**	**Melatonin group (*n* = 28)**	***P*^**a**^**
Energy (kcal/d)	2,228 ± 311	2,273 ± 300	0.58
Carbohydrates (g/d)	293.7 ± 70.1	311.7 ± 62.3	0.31
Protein (g/d)	89.1 ± 14.1	85.2 ± 12.2	0.28
Fat (g/d)	80.2 ± 11.1	79.8 ± 16.0	0.90
SFAs (g/d)	25.0 ± 5.7	24.9 ± 5.4	0.96
PUFAs (g/d)	25.4 ± 5.1	24.7 ± 6.6	0.63
MUFAs (g/d)	22.0 ± 5.0	22.4 ± 6.1	0.76
Cholesterol (mg/d)	250.6 ± 117.3	215.7 ± 101.7	0.24
TDF (g/d)	17.2 ± 4.6	18.9 ± 4.9	0.19

*Data are means ± SDs*.

a *Obtained from independent t-test*.

*MUFAs, monounsaturated fatty acids; PUFAs, polyunsaturated fatty acids; SFAs, saturated fatty acids; TDF, total dietary fiber*.

Melatonin administration significantly reduced hirsutism (β −0.47; 95% CI, −0.86, −0.09; *P* = 0.01), serum total testosterone (β −0.11 ng/mL; 95% CI, −0.21, −0.02; *P* = 0.01), hs-CRP (β −0.61 mg/L; 95% CI, −0.95, −0.26; *P* = 0.001), and plasma MDA levels (β −0.25 μmol/L; 95% CI, −0.38, −0.11; *P* < 0.001), and significantly increased plasma TAC levels (β 106.07 mmol/L; 95% CI, 62.87, 149.28; *P* < 0.001) and GSH (β 81.05 μmol/L; 95% CI, 36.08, 126.03; *P* = 0.001) compared with the placebo (Table 4). Melatonin supplementation did not affect plasma NO and serum SHBG levels.

**Table 4:** Metabolic profiles at baseline and after the 12-week intervention in women with polycystic ovary syndrome who received either melatonin supplements or placebo^a^.

**Variables**	**Placebo group (*****n*** **=** **28)**		**Melatonin group (*****n*** **=** **28)**		**Difference in outcome measures between melatonin and placebo groups**^****a****^	
	**Baseline**	**Week 12**	**Baseline**	**Week 12**	**β (95% CI)**	***P*^**b**^**
Total testosterone (ng/mL)	1.1 ± 0.3	1.1 ± 0.4	1.0 ± 0.2	0.9 ± 0.2	−0.11 (−0.21, −0.02)	0.01
SHBG (nmol/L)	39.3 ± 8.4	39.2 ± 8.3	37.7 ± 7.7	38.9 ± 8.0	1.24 (−0.31, 2.80)	0.11
mFG scores	11.5 ± 2.7	11.2 ± 2.6	12.0 ± 2.4	11.2 ± 2.3	−0.47 (−0.86, −0.09)	0.01
hs-CRP (mg/L)	4.1 ± 2.1	4.3 ± 1.9	4.3 ± 1.7	3.9 ± 1.8	−0.61 (−0.95, −0.26)	0.001
NO (μmol/L)	29.9 ± 2.9	29.5 ± 2.8	28.1 ± 2.5	28.3 ± 2.6	0.26 (−0.73, 1.26)	0.59
TAC (mmol/L)	1,011.2 ± 92.4	1,000.7 ± 100.8	1,030.0 ± 85.0	1,119.3 ± 102.7	106.07 (62.87, 149.28)	< 0.001
GSH (μmol/L)	525.7 ± 62.6	533.4 ± 63.2	568.8 ± 133.5	655.5 ± 167.9	81.05 (36.08, 126.03)	0.001
MDA (μmol/L)	2.6 ± 0.4	2.7 ± 0.5	2.6 ± 0.5	2.5 ± 0.5	−0.25 (−0.38, −0.11)	< 0.001

*Data are means ± SDs*.

a *”Outcome measures” refers to the change in values of measures of interest between baseline and week 12. β [difference in the mean outcome measures between treatment groups (melatonin group = 1 and placebo group = 0)]*.

b *Obtained from multiple-regression model (adjusted for baseline values of each biochemical variables, age, and baseline BMI)*.

*GSH, total glutathione; hs-CRP, high-sensitivity C-reactive protein; mFG, modified Ferriman–Gallwey; MDA, malondialdehyde; NO, nitric oxide; SHBG, sex hormone–binding globulin; TAC, total antioxidant capacity*.

**Figure 2:**
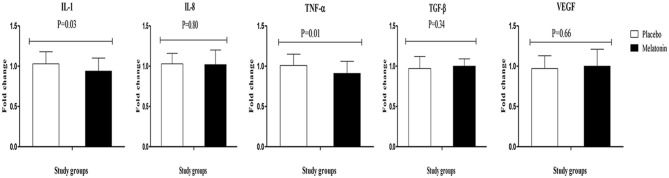
Fold change (means ± SDs) of gene expression levels for IL-1, IL-8, TNF-α, TGF-β, and VEGF were compared using independent *t*-test (*N* = 28 in each group) between women with polycystic ovary syndrome receiving melatonin supplements and those receiving placebo. IL-1, interleukin-1; IL-8, interleukin-8; PCOS, polycystic ovary syndrome; TNF-α, tumor necrosis factor alpha; TGF-β, transforming growth factor beta; VEGF, vascular endothelial growth factor.

Melatonin supplementation downregulated gene expression of IL-1 (*P* = 0.03) and TNF-α (*P* = 0.01) compared with the placebo (Figure 2). Melatonin supplementation did not influence the gene expression of IL-8, TGF-β, and VEGF.

## Discussion

In this investigation, we evaluated the impact of melatonin administration for 12 weeks on clinical status, hormonal profiles, oxidative stress biomarkers, inflammatory factors, and gene expression related to inflammation in women with PCOS who were candidates for IVF. Our findings depicted that melatonin administration for 12 weeks to women with PCOS significantly reduced hirsutism, total testosterone, hs-CRP, and MDA, while increasing TAC and GSH levels. In addition, melatonin administration reduced gene expression of IL-1 and TNF-α. Melatonin, in humans, is produced in the pineal gland, cutaneous tissue, ovary, retina, and gastrointestinal system, though its rhythmic synthesis occurs only in the pineal gland (34). Melatonin is detectable in everybody's compartment, such as in the pre-ovulatory follicular fluid, where its level is remarkably higher than that in the serum (35). Although it has been considered that the melatonin in human pre-ovulatory follicular fluid is derived from the body circulation, it may also be produced in the ovary (granulosa and oocyte cells) (36). There is current evidence demonstrating elevated melatonin concentrations in the luteal phase compared with the follicular phase of the human menstrual cycle (37). Melatonin directly stimulates the production of progesterone by granulosa cells or luteal cells and might act at the ovary level to regulate luteal function (18).

### Effects on Clinical Status and Hormonal Profiles

We found that melatonin consumption for 12 weeks in women with PCOS significantly decreased hirsutism and total testosterone levels yet did not influence SHBG values. Hyperandrogenism, the hallmark of PCOS, is a consequence of excessive androgen production by the abnormal ovarian theca cells and the augmented activity and expression of various enzymes involved in the production of steroids (2, 38). Furthermore, adrenal glands contribute to hyperandrogenism, as evidenced by raised levels of dehydroepiandrosterone sulfate (DHEAS) (39). Existing evidence suggests that decreased androgen levels are associated with an improvement in ovulatory functions, reducing hirsutism, and enhancing health-related quality of life (40, 41). It has been shown that the ovary (oocyte and granulosa cells) can produce melatonin and that melatonin receptors are detected in the membrane of human luteal and granulosa cells (20). Women with PCOS have significantly elevated melatonin concentrations and hyperandrogenemia along with an elevated number of atretic follicles (19). After 6 months of supplementation, melatonin significantly improved menstrual irregularities and biochemical hyperandrogenism in women with PCOS through a direct, insulin-independent effect on the ovary (42). In another study, melatonin administration to a PCOS mouse model potentially induced oocyte nuclear maturation and guaranteed fertilization potential (43). Melatonin is favored toward the treatment of PCOS *via* its influence on the production of steroids, thereby modulating ovulation and reducing insulin resistance and dyslipidemia (44). Overall, there are different parameters, including participants' characteristics, pancreatic beta-cell function, higher dose of melatonin, or longer period of intervention, that can provide appropriate circulating levels of melatonin necessary for improving SHBG levels. However, we were not able to assess circulating levels of melatonin in the present study. Perhaps if the dose or duration of melatonin supplementation had been increased, the results of SHBG would have been significant.

### Effects on Biomarkers of Inflammation and Oxidative Stress

Our findings showed that melatonin intake after 12 weeks in women with PCOS significantly decreased serum hs-CRP and plasma MDA, and significantly increased plasma GSH and TAC levels. Moreover, melatonin consumption downregulated IL-1 and TNF-α gene expression in PBMCs of women with PCOS, but did not affect gene expression of IL-8, TGF-β, and VEGF. Previous studies have shown a promising effect of melatonin on inflammation and oxidative stress. Mistraletti et al. (45) demonstrated that 6 mg/day melatonin administration to critically ill patients significantly ameliorated TAC levels. Melatonin intake at a dosage of 5 mg/day for 8 weeks among people with metabolic syndrome reduced MDA levels and enhanced catalase activity (46). Recently, we depicted in another study that the administration of 10 mg/day melatonin for 12 weeks to diabetic people with ischemic heart disease had favorable impacts on inflammatory status and oxidative stress parameters (26). Pakravan et al. (47) also found that supplementation with 10 mg/day melatonin significantly decreased CRP levels in people with non-alcoholic fatty liver disease. In a recent meta-analysis conducted by Akbari et al. (48), melatonin supplementation decreased systemic inflammatory markers in patients with metabolic diseases. However, melatonin consumption (6 mg/day) in obese women for 40 days did not influence hs-CRP, TAC, and MDA levels (49). The varieties in study design, population characteristics, dosage of melatonin taken, and duration of intervention might explain the discrepancies among the results of published trials. Insulin resistance might be correlated with a specific ultrasound pattern in patients with PCOS (50). In addition, increased oxidative stress and inflammatory status are correlated with an elevated risk of CVD, diabetes mellitus, hyperandrogenism, and insulin resistance in PCOS (6, 51). Both oxidative stress and PCOS have also been involved in several aspects of female reproduction (52). Melatonin can attenuate inflammatory status and oxidative damage *via* modulating the function of antioxidative enzymes, inhibiting nuclear factor-kappaB activation, enhancing glutathione synthesis, and directly scavenging free radicals in the inflamed tissues (53, 54).

This study had a few limitations. Due to a funding shortage, we were not able to measure serum melatonin and/or urinary 6-sulfatoxymelatonin (its main excretory metabolite) to assess precisely the compliance of study participants. This limitation has been considered in the interpretation of our findings. In addition, we were unable to determine the impacts of melatonin administration on other biomarkers of oxidative stress and inflammation. Also, we did not evaluate biomarkers of inflammation and oxidative stress in the ovary. Measurement of hirsutism as a clinical finding in the current study is undervalued; however, we did not evaluate menstrual cyclicity before and after the treatment, which is one of the most pivotal outcomes for the health and reproduction of women with PCOS.

Overall, this RCT suggests that melatonin administration for 12 weeks to women with PCOS might reduce hirsutism, total testosterone, hs-CRP, and MDA, while increasing TAC and GSH levels. In addition, melatonin administration might reduce gene expression of IL-1 and TNF-α.

## Ethics Statement

This randomized double-blinded, placebo-controlled trial registered in the Iranian website for registration of clinical trials (http://www.irct.ir: IRCT2017082733941N9) and followed the Declaration of Helsinki and Good Clinical Practice guidelines. The study protocol was approved by the Ethics Committee of Arak University of Medical Sciences (AUMS). Written informed consent was obtained from all participants prior to the intervention.

## Author Contributions

ZA contributed in conception, design, statistical analysis, and drafting of the manuscript. MJ, FF, EK, EsA, FB, VO, ElA, and MM contributed in data collection and manuscript drafting. All authors approved the final version for submission. ZA supervised the study. NM, MK, and TE have substantially contributed to the revised version, including checking all statistical analyses. All authors approved the final version for resubmission and change in authorship.

### Conflict of Interest Statement

The authors declare that the research was conducted in the absence of any commercial or financial relationships that could be construed as a potential conflict of interest.

## References

[1] Baptiste CG Battista MC Trottier A Baillargeon JP. Insulin and hyperandrogenism in women with polycystic ovary syndrome. J Steroid Biochem Mol Biol. (2010) 122:42–52. 10.1016/j.jsbmb.2009.12.010 20036327 PMC3846536

[2] Nelson VL Legro RS Strauss JF McAllister JM. Augmented androgen production is a stable steroidogenic phenotype of propagated theca cells from polycystic ovaries. Mol Endocrinol. (1999) 13:946–57. 10.1210/mend.13.6.0311 10379893

[3] Gilling-Smith C Story H Rogers V Franks S. Evidence for a primary abnormality of thecal cell steroidogenesis in the polycystic ovary syndrome. Clin Endocrinol. (1997) 47:93–9. 10.1046/j.1365-2265.1997.2321049.x 9302378

[4] Gonzalez F Rote NS Minium J Kirwan JP. Reactive oxygen species–induced oxidative stress in the development of insulin resistance and hyperandrogenism in polycystic ovary syndrome. J Clin Endocrinol Metab. (2006) 91:336–40. 10.1210/jc.2005-1696 16249279

[5] Gonzalez F. Inflammation in polycystic ovary syndrome: underpinning of insulin resistance and ovarian dysfunction. Steroids. (2012) 77:300–5. 10.1016/j.steroids.2011.12.003 22178787 PMC3309040

[6] Boots CE Jungheim ES. Inflammation and human ovarian follicular dynamics. Semin Reprod Med. (2015) 33:270–5. 10.1055/s-0035-1554928 26132931 PMC4772716

[7] van Zuuren EJ Fedorowicz Z Interventions for hirsutism excluding laser and therapy alone: abridged Cochrane systematic review including GRADE assessments. Br J Dermatol. (2016) 175:45–61. 10.1111/bjd.14486 26892495

[8] Reiter RJ Mayo JC Tan DX Sainz RM Alatorre-Jimenez M Qin L. Melatonin as an antioxidant: under promises but over delivers. J Pineal Res. (2016) 61:253–78. 10.1111/jpi.12360 27500468

[9] Klein DC Moore RY Pineal N-acetyltransferase and hydroxyindole-O-methyl-transferase: control by the retinohypothalamic tract and the suprachiasmatic nucleus. Brain Res. (1979) 174:245–62. 10.1016/0006-8993(79)90848-5 487129

[10] Cajochen C Kräuchi K Wirz-Justice A. Role of melatonin in the regulation of human circadian rhythms and sleep. J Neuroendocrinol. (2003) 15:432–7. 10.1046/j.1365-2826.2003.00989.x 12622846

[11] Maestroni GJ Conti A Pierpaoli W. Role of the pineal gland in immunity: circadian synthesis and release of melatonin modulates the antibody response and antagonizes the immunosuppressive effect of corticosterone. J Neuroimmunol. (1986) 13:19–30. 10.1016/0165-5728(86)90047-0 2944914

[12] Tan D-X Manchester LC Esteban-Zubero E Zhou Z Reiter RJ. Melatonin as a potent and inducible endogenous antioxidant: synthesis and metabolism. Molecules. (2015) 20:18886–906. 10.3390/molecules201018886 26501252 PMC6332205

[13] Tan D Reiter RJ Manchester LC Yan M El-Sawi M Sainz RM. Chemical and physical properties and potential mechanisms: melatonin as a broad spectrum antioxidant and free radical scavenger. Curr Top Med Chem. (2002) 2:181–97. 10.2174/1568026023394443 11899100

[14] Esposito E Cuzzocrea S. Antiinflammatory activity of melatonin in central nervous system. Curr Neuropharmacol. (2010) 8:228–42. 10.2174/157015910792246155 21358973 PMC3001216

[15] Ecklund LC Usadi RS. Endocrine and reproductive effects of polycystic ovarian syndrome. Obstet Gynecol Clin North Am. (2015) 42:55–65. 10.1016/j.ogc.2014.09.003 25681840

[16] Thessaloniki ESHRE/ASRM-Sponsored PCOS Consensus Workshop Group Consensus on infertility treatment related to polycystic ovary syndrome. Hum Reprod. (2008) 23:462–77. 10.1093/humrep/dem426 18308833

[17] Cagnacci A Volpe A. A role for melatonin in PCOS? Fertil Steril. (2002) 77:1089. author reply:90. 10.1016/S0015-0282(02)03070-4 12009381

[18] Tamura H Nakamura Y Korkmaz A Manchester LC Tan DX Sugino N. Melatonin and the ovary: physiological and pathophysiological implications. Fertil Steril. (2009) 92:328–43. 10.1016/j.fertnstert.2008.05.016 18804205

[19] Jain P Jain M Haldar C Singh TB Jain S. Melatonin and its correlation with testosterone in polycystic ovarian syndrome. J Hum Reprod Sci. (2013) 6:253–8. 10.4103/0974-1208.126295 24672165 PMC3963309

[20] Yang HL Zhou WJ Gu CJ Meng YH Shao J Li DJ. Pleiotropic roles of melatonin in endometriosis, recurrent spontaneous abortion, and polycystic ovary syndrome. Am J Reprod Immunol. (2018) 80:e12839. 10.1111/aji.12839 29493042

[21] Shreeve N Cagampang F Sadek K Tolhurst M Houldey A Hill CM. Poor sleep in PCOS; is melatonin the culprit? Hum Reprod. (2013) 28:1348–53. 10.1093/humrep/det013 23438443

[22] Spinedi E Cardinali DP. The polycystic ovary syndrome and the metabolic syndrome: a possible chronobiotic–cytoprotective adjuvant therapy. Int J Endocrinol. (2018) 2018:1349868. 10.1155/2018/1349868 30147722 PMC6083563

[23] Pacchiarotti A Carlomagno G Antonini G Pacchiarotti A. Effect of myo-inositol and melatonin versus myo-inositol, in a randomized controlled trial, for improving *in vitro* fertilization of patients with polycystic ovarian syndrome. Gynecol Endocrinol. (2016) 32:69–73. 10.3109/09513590.2015.1101444 26507336

[24] Unfer V Raffone E Rizzo P Buffo S. Effect of a supplementation with myo-inositol plus melatonin on oocyte quality in women who failed to conceive in previous *in vitro* fertilization cycles for poor oocyte quality: a prospective, longitudinal, cohort study. Gynecol Endocrinol. (2011) 27:857–61. 10.3109/09513590.2011.564687 21463230

[25] Rotterdam ESHRE/ASRM-Sponsored PCOS Consensus Workshop Group Revised 2003 consensus on diagnostic criteria and long-term health risks related to polycystic ovary syndrome. Fertil Steril. (2004) 81:19–25. 10.1016/j.fertnstert.2003.10.004 14711538

[26] Raygan F Ostadmohammadi V Bahmani F Reiter RJ Asemi Z. Melatonin administration lowers biomarkers of oxidative stress and cardio-metabolic risk in type 2 diabetic patients with coronary heart disease: a randomized, double-blind, placebo-controlled trial. Clin Nutr. (2019) 38:191–6. 10.1016/j.clnu.2017.12.004 29275919

[27] Jamilian M Foroozanfard F Bahmani F Talaee R Monavari M Asemi Z. Effects of zinc supplementation on endocrine outcomes in women with polycystic ovary syndrome: a randomized, double-blind, placebo-controlled trial. Biol Trace Elem Res. (2016) 170:271–8. 10.1007/s12011-015-0480-7 26315303

[28] Hatch R Rosenfield RL Kim MH Tredway D. Hirsutism: implications, etiology, and management. Am J Obstet Gynecol. (1981) 140:815–30. 10.1016/0002-9378(81)90746-8 7258262

[29] Ramezani Tehrani F Minooee S Azizi F Validation of a simplified method to assess hirsutism in the Iranian population. Eur J Obstet Gynecol Reprod Biol. (2014) 174:91–5. 10.1016/j.ejogrb.2013.12.008 24393448

[30] Tatsch E Bochi GV Pereira Rda S Kober H Agertt VA de Campos MM. A simple and inexpensive automated technique for measurement of serum nitrite/nitrate. Clin Biochem. (2011) 44:348–50. 10.1016/j.clinbiochem.2010.12.011 21185277

[31] Benzie IF Strain JJ. The ferric reducing ability of plasma (FRAP) as a measure of antioxidant power: the FRAP assay. Anal Biochem. (1996) 239:70–6. 10.1006/abio.1996.0292 8660627

[32] Beutler E Gelbart T. Plasma glutathione in health and in patients with malignant disease. J Lab Clin Med. (1985) 105:581–4. 3989350

[33] Dunkley PR Jarvie PE Robinson PJ. A rapid Percoll gradient procedure for preparation of synaptosomes. Nat Protoc. (2008) 3:1718–28. 10.1038/nprot.2008.171 18927557

[34] Cassone VM Natesan AK. Time and time again: the phylogeny of melatonin as a transducer of biological time. J Biol Rhythms. (1997) 12:489–97. 10.1177/074873049701200602 9406022

[35] Nakamura Y Tamura H Takayama H Kato H Increased endogenous level of melatonin in preovulatory human follicles does not directly influence progesterone production. Fertil Steril. (2003) 80:1012–6. 10.1016/S0015-0282(03)01008-2 14556825

[36] Ronnberg L Kauppila A Leppaluoto J Martikainen H Vakkuri O. Circadian and seasonal variation in human preovulatory follicular fluid melatonin concentration. J Clin Endocrinol Metab. (1990) 71:492–6. 10.1210/jcem-71-2-493 2380343

[37] Wetterberg L Arendt J Paunier L Sizonenko PC Donselaar W Heyden T. Human serum melatonin changes during the menstrual cycle. J Clin Endocrinol Metab. (1976) 42:185–8. 10.1210/jcem-42-1-185 1249188

[38] Wickenheisser JK Quinn PG Nelson VL Legro RS Strauss JF McAllister JM. Differential activity of the cytochrome P450 17alpha-hydroxylase and steroidogenic acute regulatory protein gene promoters in normal and polycystic ovary syndrome theca cells. J Clin Endocrinol Metab. (2000) 85:2304–11. 10.1210/jcem.85.6.6631 10852468

[39] Kumar A Woods KS Bartolucci AA Azziz R. Prevalence of adrenal androgen excess in patients with the polycystic ovary syndrome (PCOS). Clin Endocrinol. (2005) 62:644–9. 10.1111/j.1365-2265.2005.02256.x 15943823

[40] Dokras A Sarwer DB Allison KC Milman L Kris-Etherton PM Kunselman AR. Weight loss and lowering androgens predict improvements in health-related quality of life in women with PCOS. J Clin Endocrinol Metab. (2016) 101:2966–74. 10.1210/jc.2016-1896 27253669 PMC4971336

[41] Boztosun A Acmaz G Ozturk A Muderris II. Clinical efficacy of low dose flutamide plus Diane-35 in the treatment of idiopathic hirsutism and polycystic ovary syndrome. Ginekol Pol. (2013) 84:258–62. 10.17772/gp/1573 23700857

[42] Tagliaferri V Romualdi D Scarinci E Cicco S Florio CD Immediata V. Melatonin treatment may be able to restore menstrual cyclicity in women with PCOS: a pilot study. Reprod Sci. (2018) 25:269–75. 10.1177/1933719117711262 28558523

[43] Nikmard F Hosseini E Bakhtiyari M Ashrafi M Amidi F Aflatoonian R. Effects of melatonin on oocyte maturation in PCOS mouse model. Anim Sci J. (2017) 88:586–92. 10.1111/asj.12675 27530294

[44] Pai SA Majumdar AS. Protective effects of melatonin against metabolic and reproductive disturbances in polycystic ovary syndrome in rats. J Pharm Pharmacol. (2014) 66:1710–21. 10.1111/jphp.12297 25176048

[45] Mistraletti G Paroni R Umbrello M D'Amato L Sabbatini G Taverna M. Melatonin pharmacological blood levels increase total antioxidant capacity in critically Ill patients. Int J Mol Sci. (2017) 18:759. 10.3390/ijms18040759 28368352 PMC5412344

[46] Kozirog M Poliwczak AR Duchnowicz P Koter-Michalak M Sikora J Broncel M. Melatonin treatment improves blood pressure, lipid profile, and parameters of oxidative stress in patients with metabolic syndrome. J Pineal Res. (2011) 50:261–6. 10.1111/j.1600-079X.2010.00835.x 21138476

[47] Pakravan H Ahmadian M Fani A Aghaee D Brumanad S Pakzad B. The effects of melatonin in patients with nonalcoholic fatty liver disease: a randomized controlled trial. Adv Biomed Res. (2017) 6:40. 10.4103/2277-9175.204593 28503495 PMC5414412

[48] Akbari M Ostadmohammadi V Tabrizi R Lankarani KB Heydari ST Amirani E The effects of melatonin supplementation on inflammatory markers among patients with metabolic syndrome or related disorders: a systematic review and meta-analysis of randomized controlled trials. Inflammopharmacology. (2018) 26:899–907. 10.1007/s10787-018-0508-7 29907916

[49] Mesri Alamdari N Mahdavi R Roshanravan N Lotfi Yaghin N Ostadrahimi AR Faramarzi E A double-blind, placebo-controlled trial related to the effects of melatonin on oxidative stress and inflammatory parameters of obese women. Horm Metab Res. (2015) 47:504–8. 10.1055/s-0034-1384587 25126957

[50] Alviggi C Conforti A De Rosa P Strina I Palomba S Vallone R. The distribution of stroma and antral follicles differs between insulin-resistance and hyperandrogenism-related polycystic ovarian syndrome. Front Endocrinol. (2017) 8:117. 10.3389/fendo.2017.00117 28620353 PMC5449504

[51] Pawelczak M Rosenthal J Milla S Liu YH Shah B. Evaluation of the pro-inflammatory cytokine tumor necrosis factor-alpha in adolescents with polycystic ovary syndrome. J Pediatr Adolesc Gynecol. (2014) 27:356–9. 10.1016/j.jpag.2014.01.104 25256873 PMC4536070

[52] Alviggi C Cariati F Conforti A De Rosa P Vallone R Strina I. The effect of FT500 Plus((R)) on ovarian stimulation in PCOS women. Reprod Toxicol. (2016) 59:40–4. 10.1016/j.reprotox.2015.10.014 26545973

[53] Lin YW Lee LM Lee WJ Chu CY Tan P Yang YC. Melatonin inhibits MMP-9 transactivation and renal cell carcinoma metastasis by suppressing Akt-MAPKs pathway and NF-kappaB DNA-binding activity. J Pineal Res. (2016) 60:277–90. 10.1111/jpi.12308 26732239

[54] Mohan N Sadeghi K Reiter RJ Meltz ML. The neurohormone melatonin inhibits cytokine, mitogen and ionizing radiation induced NF-kappa B. Biochem Mol Biol Int. (1995) 37:1063–70. 8747536

